# SARS-CoV-2 infection and psychiatric manifestations in a previous healthy patient

**DOI:** 10.22088/cjim.11.0.566

**Published:** 2020

**Authors:** Noel Lorenzo-Villalba, Xavier Jannot, Anezka Syrovatkova, Vincent Michel, Emmanuel Andrès

**Affiliations:** 1 Department of Internal Medicine, Strasbourg University, Strasbourg, France

**Keywords:** SARS infection, hallucinations, unusual behavior, psychosis

## Abstract

**Background:**

The clinical presentation of SARS-CoV-2 infection was initially dominated by respiratory symptoms. However, the clinical spectrum is wide and neuropsychiatric syndromes are also a source of medical concern. Our aims are to present an atypical clinical presentation of SARS-CoV-2 infection characterized by auditory hallucinations and unusual behavior and to emphasize the diversity of clinical manifestations of SARS-CoV-2 infection.

**Case Presentation:**

A 33-year-old woman was admitted to the emergency department (ED) with a one-day history of auditory hallucinations, unusual behavior, changes in her sleeping habits and incoherent speech. No other symptoms were reported. Blood examinations confirmed high elevated white cell count and C-reactive protein. The head CT scan was normal but the chest scan showed right ground-glass opacities in the lower zones. The oropharyngeal swab was positive for SARS-CoV-2**. **Based on these results, the diagnosis of SARS-CoV-2 infection was retained. The patient received no specific treatment for SARS-CoV-2 infection and only needed oxygen therapy support for 7 days. The additional dose of Olanzapine 10 mg daily was initially prescribed but the patient was back to her usual self on day 14 of hospital admission leading to its discontinuation. This clinical course was consistent with a first episode of psychosis triggered by SARS-CoV-2 infection.

**Conclusion:**

Neuroinflammation owing to SARS-CoV-2 infection could be responsible for a wide and unknown spectrum of neuropsychiatric manifestations. During this pandemic, special attention should be given to patients with no previous history of psychiatric disorders presenting to ED with neuropsychiatric syndromes of unknown etiology.

Neuropsychiatric syndromes affecting cognitive, behavioural and perceptual domains have been related to the CNS invasion in viral infections (1). In this respect, some studies report the presence of coronaviruses in both the brain and the cerebrospinal fluid of individuals with seizures, encephalitis, and encephalomyelitis (2). The neuropsychiatric manifestations could result from the brain damage owing to direct virus toxic effects or as a consequence of the immune response or therapy. The significant release of interleukins and chemokines in severe forms of Covid-19 infected patients are associated to neuroinflammation and brain damage (1). We report here an atypical case presentation of SARS-CoV-2 infection characterized by auditory hallucinations and unusual behavior in the absence of fever of respiratory symptoms.

## Case presentation

A 33-year-old woman was admitted to the emergency department (ED) with a one-day history of auditory hallucinations and unusual behavior (she was found naked in the building basement). 

Their relatives also referred she had changed her sleeping habits and that her language did not make sense. No fever, cough, shortness of breath, weight loss, or gastrointestinal symptoms were reported. She had no significant medical history, was not taking any medications, and did not have any allergies. She did occasionally drink alcohol, but no history of tobacco smoking or illicit drugs using was reported. The patient has not traveled abroad in the past 6 months and she has had no contact with any known sick persons. Her family history was irrelevant 

The physical examination showed a patient alert and oriented in person but her speech was incoherent, she referred hearing voices around her in the room and she stated being in a castle despite our efforts to make her realize she was in a medical room. Her blood pressure was 120/80 mmHg with a regular heart rate ranging between 90-100 beats/min. Her respirations were 20 breaths/min, her oral temperature was 36.7°C, and her oxygen saturation was 94% on room air. Heart sounds were normal with a regular rhythm and lung fields were clear. Her abdomen was soft and painless, no abdominal masses or hepatosplenomegaly were appreciated. Upon neurologic examination, she became fully awake and had no neck stiffness or any other signs of meningeal irritation. Her pupils were round, mid-sized, and reactive to light bilaterally. No neurologic deficits were found.

**Figure 1:**
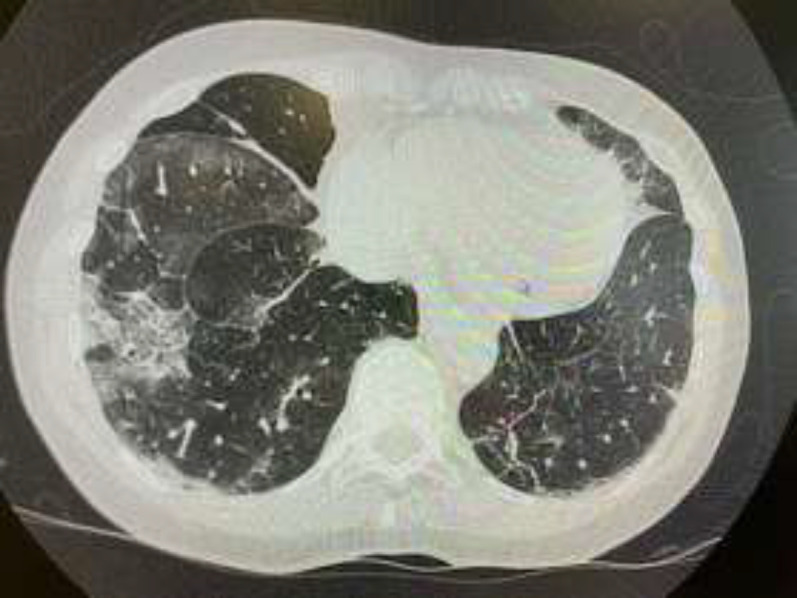
Chest CT scan showing right lower pulmonary opacity

A complete blood count and comprehensive metabolic panel show abnormalities in the white blood cell (WBC) count of 22.3×10^3^ cells/µL (3.90-10.50×10^3^ cells/µL), band neutrophils of 15×10^3^cells/µL(1.80-7.90×10^3^cells/µL), and C-reactive protein of 98 mg/L(<4.0 mg/L). Electrolytes, kidney, liver and thyroid function tests were within the normal range. Arterial blood gas analysis showed a pH of 7.38(7.35-7.45), a pCO_2_ of 35 mmHg (35-45 mmHg), a pO_2_ of 72 mmHg (83-108 mmHg), a bicarbonate level of 24 mmol/L(20-27 mmol/L) so oxygen supplementation was initiated. Toxics in blood and urine were negative. An urgent plain head CT scan was performed but came back normal. The investigations were completed with chest computed tomography (CT) showing right ground-glass opacity consistent with SARS-CoV-2 infection (figure 1). The oropharyngeal swab for Covid-19 testing was positive.

The patient was hospitalized with the presumptive diagnosis of first episode of psychosis (FEP) probably triggered by SARS-CoV-2 infection. The patient was admitted to the internal medicine department. Because of lack of evidence-based medicine validated therapy for SARS-CoV2 infection, the patient did not benefit from any specific treatment, antibiotics were not considered and only oxygen therapy with 2L/min via nasal cannulation was needed during 7 days. Additional dose of Olanzapine 10 mg daily was initially prescribed to control the psychiatric syndrome but the patient was completed back to her usual self on day 14 of hospital admission. As the result of medical consensus, Olanzapine was discontinued. The patient was discharged on day 14 and a follow up appointment at the psychiatry outpatient clinic was set up three months later. 

## Discussion

Reports about the clinical presentation of SARS-CoV-2 infection were initially dominated by respiratory symptoms and less commonly gastrointestinal manifestations. However, the virus invasion is not only limited to these systems as the result of the significant expression of ACE2 in other tissues like kidney, endothelium, heart, and central nervous system (CNS). In this sense, the viral invasion of the central nervous system may lead to multiple neurological and psychiatric consequences (1, 3). The SARS-CoV-2 could reach the CNS through the axons of the olfactory nerve or through endothelial cells lining brain vasculature (1, 4).

The significant release of interleukins and chemokines is associated with systemic inflammation and lymphopenia. In patients developing severe forms of COVID-19 is related to a prolonged viral load persistence and reactive gliosis which perpetuate neuroinflammation (1, 5). Neuroinflammation is well known to be related to multiple neuropsychiatric and neuro-cognitive disorders such as psychosis, depression, sleep disorders and others (1, 6). However, the neuropsychiatric impact of this pandemic is still not known. 

A significant increase in pro-inflammatory cytokines and their receptors have been described in both chronic schizophrenia and first episode of psychosis. Regarding IL-6, increased levels have been detected in cerebrospinal fluid patients with schizophrenia and its high levels in young individuals have also been related to the development of psychosis (1). 

Besides, an upregulation of IL-6, TNF-α and IL-1β was found in FEP patients compared with healthy siblings. This fact suggests that familiar vulnerability is not associated with inflammatory-related psychotic reactions (7, 8). In this case, drug‐induced psychosis was ruled out through drug screening and proper history. This entity also represents a challenge in the differential diagnosis as it may present with features overlapping reactive psychosis such as an acute or abrupt onset, fragmentary psychotic symptoms and fluctuating clouded consciousness.

In conclusions Neuroinflammation owing to SARS-CoV-2 infection could be responsible for a wide and still unknown spectrum of neuropsychiatric manifestations. During this pandemic period, special attention should be given to patients with no previous history of psychiatric disorders presenting to ED with neuropsychiatric syndromes of unknown etiology.

## Ethic consideration:

Written consent from the patient was available. Internal Department Ethics Committee approved this paper for publication (N°22-11-20). 
